# Validation of the Retirement Resources Inventory in Persian: Assessing Psychometric Properties among Iranian Retirees

**DOI:** 10.1155/2024/1467773

**Published:** 2024-08-26

**Authors:** Elaheh Shoushtari-Moghaddam, Abdolrahim Asadollahi, Mohammad Hossein Kaveh

**Affiliations:** ^1^ Student Research Committee Department of Health Education and Promotion School of Health Shiraz University of Medical Sciences, Shiraz, Iran; ^2^ Department of Gerontology School of Health Shiraz University of Medical Sciences, Shiraz, Iran; ^3^ Research Center for Health Sciences Institute of Health Department of Health Education and Promotion School of Health Shiraz University of Medical Sciences, Shiraz, Iran

## Abstract

**Introduction:**

Retirement resources are considered one of the key determinants of well-being and coping with the challenging situations of retirement courses. Therefore, due to the lack of a valid questionnaire for research and practice at the national level as well as international comparisons, this study was conducted to translate the original retirement resources inventory into Persian and assess the validity and reliability of the Persian version.

**Materials and Methods:**

The research employed a cross-sectional descriptive methodological approach, beginning with translating the questionnaire into Persian. Psychometric properties were evaluated through face, content, construct validity, and reliability tests. 335 participants were involved in assessing construct validity, while 30 participants contributed to the internal consistency test, and 20 took part in the test-retest reliability test. Data analysis was performed using SPSS Version 26 and AMOS 24 software.

**Results:**

The exploratory factor analysis (EFA) revealed eight components with eigenvalues greater than 1, accounting for 46.68% of the variance, with no questions being removed. Confirmatory factor analysis (CFA) indicated that all items had a factor loading above 0.3, resulting in a final model with 35 items and eight factors, supported by fit indices (*χ*^2^/df = 1.95, TLI: 0.87, IFI = 0.88, RMSEA = 0.05, GFI: 0.85). Reliability measures showed Cronbach's alpha coefficients ranging from 0.71 to 0.83 and an intraclass correlation coefficient (ICC) of 0.93.

**Conclusion:**

The study concludes that the Persian version of the RRI, encompassing 35 items across eight dimensions, is a valid and reliable tool for the Iranian retiree population. This validated inventory can be utilized in future national and international studies, particularly experimental ones, to develop well-being and retirement adjustment programs, thereby aiding in understanding and supporting Iranian retirees.

## 1. Introduction

The growing population of older adults in underdeveloped countries faces numerous challenges, particularly concerning retirement needs, potentially leading to financial deficits and social risks that impact physical health and quality of life [1]. These challenges are exacerbated by inadequate social security systems, limited access to healthcare, and the lack of pension plans, leaving many older adults financially vulnerable [2]. Changes in physical, mental, and social functions pose significant adjustment difficulties for retirees [3]. The absence of formal support networks and community resources further compounds their struggles [4], highlighting the urgency of implementing comprehensive policies and programs to support their retirement needs. Effective retirement adjustment is crucial in addressing these issues, ensuring that retirees can maintain their physical and mental well-being during this significant life event [5].

Retirement adjustment involves adapting to transition-induced changes and reflects an individual's mental comfort with post-retirement life. Accessing valuable resources is crucial for retirees to adjust to their new status outside the workplace [6]. Hence, we could define retirement adjustment as the process through which retirees adapt to changes in their roles, routines, and resources post-retirement, highlighting the multidimensional nature of retirement resources encompassing physical, financial, social, affective, cognitive, and motivational aspects [7].

Hobfoll (2002) defined resources as the total capacity for fulfilling an individual's valued needs and goals. According to his conservation of resources theory, retirement resources encompass various essential resources crucial for retirement adjustment [8]. The resource-based dynamic model categorizes these resources into six types: physical (e.g., health status), financial (e.g., pension, savings), social (e.g., social network), affective (e.g., emotions), cognitive (e.g., perceived control), and motivational (e.g., adaptability) [9]. These resources facilitate retirement adjustment and influence retirees' well-being [10, 11].

Pre-retirement circumstances like financial status and work commitment influence these resources during retirement [12]. The model suggests three adjustment outcomes: maintaining well-being, experiencing distress from resource deficits, or positively adapting to additional resources [9]. The key tenet is that retirees' access to resources directly influences their well-being and adaptation outcomes. Studies across diverse cultural contexts corroborate the positive association between retirement resources and well-being.

For instance, Pilehvari et al. found that social networks significantly enhance the well-being of retirees in the United States [13]. Similarly, a study by Topa highlighted the importance of financial resources for retirement satisfaction in European countries [14]. Additionally, a cross-cultural study by Wang and Hesketh demonstrated that access to health resources is critical for retirement well-being in China [15]. Kaveh et al. found that better retirement resources improve adjustment and quality of life in Iran [16]. These studies underscore the universal importance of retirement resources for adjustment and quality of life, irrespective of cultural context.

Validly and reliably measuring retirement resources is crucial for scientific research and intervention planning to enhance retirement health. Wang et al. designed an instrument with a six-factor model to measure retirement resources [9], after which Leung and Earl evaluated the six types of resources and categorized them into three factors: mental resources (RT1), social resources (RT2), and physical-financial resources (RT3). The factor analysis's results showed that the goodness-of-fit of the three-factor model was better than that of the six-factor model which was originally proposed by Wang et al. The Retirement Resources Inventory (RRI), developed by Leung and Earl, offers a comprehensive approach with good psychometric properties to assessing retirement resources [17]. Their findings aid in identifying essential resources for retirement planning and guide practitioners in developing tailored interventions to address resource deficiencies among retirees. The RRI demonstrates high internal reliability, with a Cronbach's alpha value of 0.89 [17]. It has been validated in various countries and languages, including Hong Kong [11], Egypt [18], and Brazil [19], with Cronbach's alpha values ranging from 0.85 to 0.90, affirming its robustness across diverse cultural contexts.

The absence of a Persian version of the RRI is notable, considering Persian's status as the official language of three countries, including Iran, and its widespread usage among native speakers and immigrants across various nations. Iran is a combination of ethnicities with different cultures and languages, including Persians, Turks, Arabs, Baloch, and Kurds. The majority of Iran's population is made up of Muslims, but religious minorities such as Zoroastrians, Jews, and Christians also live there. However, the Persian language and ancient Iranian culture and customs are common to all ethnic groups. With Iran's population surpassing 84 million [20], the availability of a validated Persian version of the inventory would significantly aid researchers both nationally and internationally. Hence, this study aims to translate and validate the RRI into Persian to provide researchers and practitioners with a reliable tool for assessing retirement resources among Iranian retirees, contributing to the enhancement of retirement health and well-being.

## 2. Materials and Methods

### 2.1. Participants and Sampling

The participants in the research were retired members of the retirement centers of the big universities of Isfahan in the center and Shiraz in the south of the country, which are part of the big cities and because they were accessible to the research team, were chosen as research locations.

Since the implementation phase of this study coincided with the COVID-19 pandemic, two approaches were used for sampling and data collection. First, the questionnaire was sent electronically to potential participants through virtual social networks in which retirees were members as a group. Secondly, the snowball technique (inviting other retirees by the initial participants) was used to complete the sampling process; if they met the inclusion criteria, they were included in the study. The recruitment was performed from November 2021 to April 2022.

The retirees with at least one year of retirement duration, receiving a salary from one of the pension funds, having a smartphone, and being members of at least one social media platform were included in the study. Retirees who failed to complete the questionnaire properly did not return it or for any reason refused to cooperate with the project were excluded.

A sample size of 332 was determined based on the Pass section of the NCSS sample size determination software (version 15) [21], which was increased to 369 to ensure and prevent sample loss, with CI = 5 and CL = 95% in the statistical population of 1000 people. Finally, 335 questionnaires were analyzed.

### 2.2. Analysis of Data

The data were analyzed by exploratory factor analysis (EFA), confirmatory factor analysis (CFA), and ROC analysis using SPSS V.26 [22] and AMOS V.24 [23]. The significance level was set at *p* ≤ 0.05.

### 2.3. Design

This was a methodological study with a cross-sectional design. The study was conducted in two phases. Translation and adaptation of the RRI to Persian were carried out during the 1st phase. The psychometric properties of the Persian version of the RRI were analyzed in the 2nd phase.

### 2.4. Phase 1: Translation and Adaptation

The translation and adaptation of the RRI followed the World Health Organization (WHO) process [24]. Permission for a Persian translation was obtained from Joanne Earl (Professor, School of Psychological Sciences Center for Aging, Cognition, and Wellbeing, https://orcid.org/0000-0002-0232-053X). Two experts, health promotion professionals, and gerontologists, translated the RRI into Persian. The translated scale was back-translated into English by two bilingual independent translators (general practitioner and epidemiologist), who did not know the questionnaire, and the back-translated version was compared to the original for content, semantics, and concepts. An expert panel, comprising an English expert and two specialists in health promotion, one geriatric, and one psychology, reviewed the translation for equivalence and clarity. To ensure cultural adaptation, explanatory sentences were converted into question forms, response scales were standardized, and rephrasing was done. Face validity was assessed using a convenience sample of 10 retirees, resulting in good face validity for the Persian version.

The expert panel members were selected by the research team from among those who were academic staff and by reviewing their resumes and expertise.

The final translated Persian version was tested in a pilot test among 30 retirees having inclusion criteria by using the convenience sampling method. In the pilot version, Cronbach's alpha coefficient was 0.92, indicating that it is acceptable for internal consistency [25].

### 2.5. Phase 2: Psychometric Testing

The psychometric properties tested included validity and reliability. Using Cronbach's alpha coefficients, we tested the consistency of the overall scale and all the subscales. A structural validity test was carried out using EFA.

### 2.6. Content Validity

Content validation was conducted quantitatively and qualitatively based on an expert panel review to ensure that the items adequately covered the construct domains. To ensure that the items measured the intended construct and also cultural adaptation, the tool's content validity index (CVI) and content validity ratio (CVR) were assessed based on the opinion of an expert panel (15 members) composed of experts and researchers who were familiar with the methodology of psychometric testing (health promotion professionals, gerontologists, and psychologists). As an open question, we asked the experts to comment if an item needs editing or any other changes.

To determine the CVR, the experts were asked to evaluate the necessity of each item using a three-point rating scale: “Essential,” “Useful, but not essential,” or “Not necessary,” calculated by using the following formula: CVR = (ne − N/2)/(N/2) [26]. The Lawshe table indicates that in the case of assessments by 10 experts or more, the minimum agreed value for the CVR should be greater than 0.62 [27]. By asking experts to rate each item based on its relevance, simplicity, and clarity, the CVI has been assessed [28] on a scale from 1 = not relevant, simple, or clear to 4 = very relevant, simple, and clear. The CVI was calculated by the proportion of items that received a rating between 3 and 4 on the questionnaire. Waltz and Bausell's method was used to determine the CVI. According to this method, a CVI < 0.7 is unacceptable, a CVI < 0.78 requires modification and revision, and a CVI ≥ 0.79 is acceptable [29]. To calculate the CVI, the experts who rated the item as 3 or 4 were divided by the total number of experts.

### 2.7. Construct Validity

The assessment of the appropriateness of the 35 items of the RRI was carried out using EFA. EFA can be used to determine which theoretical constructs lie under a given dataset and the extent to which these constructs represent the original variables [30]. To determine sampling adequacy and the appropriateness of the factor analysis, Bartlett's test of sphericity and the Kaiser–Meyer–Olkin test were used. To extract latent factors and appropriate items from the factors, eight rotation methods and six rotation methods were tested. However, the unweighted least squares method with quartimax rotation and Kaiser normalization rotation was a theoretically and statistically better model. Each item was assigned to a factor based on an absolute value of 0.3 [31]. According to Earl's study, 3 factorial models were tested, but the total variance index was less than 40%, which could not be relied upon [32].

Then, confirmatory factor analysis (CFA) was performed using AMOS 24. CFA was subsequently employed to validate the structure by testing its fit to a hypothesized model, ensuring that the constructs identified by EFA accurately reflect the data [30]. AMOS was utilized due to its robust capability in structural equation modeling (SEM), which allows for the simultaneous estimation of multiple relationships between observed and latent variables [33]. A model has therefore been designed, and fit indices based on the cutoff values have been reported.

### 2.8. Reliability

To verify the reliability of this scale, internal consistency using Cronbach's alpha coefficient and the coefficient of correlation between the interitem and item-total correlation coefficients were calculated. An alpha value between 0.70 and 0.95 was considered acceptable [34]. The test-retest reliability was also considered, using the intraclass correlation coefficient (ICC) for 20 retirees with an interval of two weeks. An ICC greater than 0.70 indicated adequate stability, less than 0.3 indicated weak stability, and between 0.30 and 0.70 indicated moderate and acceptable stability [35].

### 2.9. ROC Analysis

To determine the best cutoff point, receiver operating characteristic (ROC) curve analysis was performed using SPSS version 26. ROC analysis was conducted to evaluate the diagnostic performance of the questionnaire, determining its ability to accurately distinguish between different outcome groups [36]. This helps assess the sensitivity and specificity of the questionnaire, ensuring its effectiveness in identifying the target constructs within the population.

### 2.10. Tool

#### 2.10.1. Retirement Resources Inventory (RRI)

The inventory was developed by Leung and Earl and includes 35 questions in three categories—physical-financial (items 1–8), social (items 9–17), and mental (items 18–35)—with a 5-point Likert scale. The range of scores is from 35 to 175. Higher scores represent more resources possessed by the retirees. Results of the CFA showed the goodness-of-fit using Time 1 data [AIC = 8289.903; BIC = 8604.791; *χ*^2^(515) = 819.722; CFI = 0.828, RMSEA = 0.071], [AIC = 8912.956; BIC = 9252.804; *χ*^2^(579) = 1014.160; CFI = 0.770, RMSEA = 0.080], Δ*χ*^2^(64) = 194.438, *p* < 0.00. Cronbach's alpha was 0.89 due to the high internal reliability of this questionnaire [17].

#### 2.10.2. Ethical consideration

Ethical approval was granted by the Ethics Committee of the Shiraz University of Medical Science (Ref. no: IR.SUMS.SCHEANUT.REC.1400.024). The participants were sent an information note with the questionnaire explaining the study's purposes, confidentiality, and their right to withdraw at any time without consequences. Since the data were collected via social networks, answering the questionnaire indicated their informed consent to participate in the study. All retirees participated in the study voluntarily, without any compulsion. Considering confidentiality, data were collected anonymously and no individual information is included in the published results. The study was based on the Helsinki Convention (2013) as well as the STROBE checklist [37].

## 3. Results

### 3.1. Participant Description

The retirees (46.3% female and 53.4% male) had a mean age of 64.2 ± 6.8 years, and the most frequent education level was a bachelor's degree or above (50.8%). Approximately 80.6% were married, 51.3% had retired in the last 10 years, and only 20.3% had a job after retirement. Most retired individuals had worked for 30 years and were required to retire (63.6%), and 58.2% lived in families of 3-4 members. Most participants were from the Isfahan University of Medical Sciences (31.9%). The majority of the participants were retired from the administrative part of the universities (33.4%), and only 13.7% of the participants were academic staff members (Table 1).

### 3.2. Content Validity

The two measures of content validity, the CVI (0.93) and the CVR (0.87) index, were good. Therefore, the number of items in the questionnaire (number = 35) was similar to that in the original questionnaire.

### 3.3. Construct Validity

#### 3.3.1. Exploratory Factor Analysis

The results of Bartlett's test of sphericity (*χ*^2^ = 4641.43; *p* < 0.001) and the Kaiser–Meyer–Olkin test (KMO = 0.87) revealed the capability of the correlation matrix and provided minimum standards for conducting a factor analysis [38]. Therefore, the 35-item questionnaire was subjected to unweighted least squares and quartimax rotation with 6 Kaiser normalization rotations, and 6 factors were identified. They were categorized as follows: F1 & F6 = mental resources (18 items); F2 = structural social support resources (6 items); F3 = financial resources (4 items); F4 = physical resources (4 items); and F5 = received social support resources (3 items) (Table 2).

Because the items under factors 1 and 6 seemed conceptually different and evoked certain psychological concepts, we analyzed these factors separately. Conducting EFA twice allowed for a more nuanced exploration of the underlying factor structure of the questionnaire, with the first analysis focusing on overall factor structure and the second analysis refining the factors based on conceptual similarities. Therefore, the items of these factors were placed under 4 factors. As a result, they were categorized under the following headings: emotional, cognitive, personal agency, and self-control. For this factor analysis, Unweighted Least Squares and Rotation Method: Equamax with Kaiser normalization with 9 rotations and an absolute value <0.3 was used (Table 3).

The choice of quartimax rotation for the first EFA was based on its ability to simplify the rows of the factor loading matrix, which helps in identifying the primary factors by reducing the complexity of each variable's loadings. Equamax rotation was then used for the second EFA to balance the simplification of both rows and columns, providing a more balanced and interpretable factor structure [40].

Therefore, in general, the final structure of the RRI, including its subfactors and related items, is shown in Table 4.

#### 3.3.2. Confirmatory Factor Analysis

**Figure 1:**
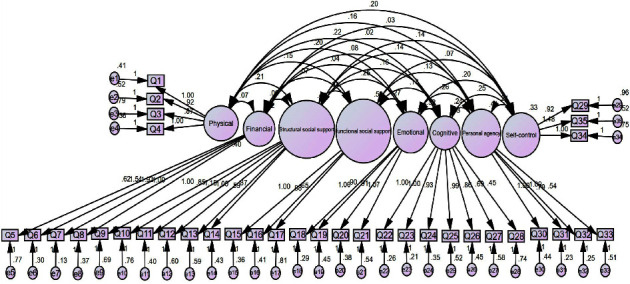
Confirmatory factor analysis of the RRI eight-factor model.

The CFA for the RRI's eight-factor model is presented in Figure 1. Each item had a statistically significant loading on its latent factor (<0.001). According to the results of Table 5, the model fit indices were as follows: TLI = 0.87, CFI = 0.88, RMSEA = 0.05, and SRMR = 0.05.

#### 3.3.3. Reliability

Cronbach's alpha was 0.90 for the whole scale, and the ICC obtained with a confidence interval of 0.95 was 0.93, all of which are statistically significant (*p* ≤ 0.0001). Cronbach's alpha coefficients of eight subscales, namely, physical (0.73), financial (0.74), structural social support (0.79), functional social support (0.71), emotional (0.79), cognitive (0.83), personal agency (0.75), and self-control (0.70), were all at favorable levels.

#### 3.3.4. Scoring, Receiver Operating Characteristic Curve, and Cut-Off Point

The sum of points per item is represented by the overall score. The item score ranged between 1 and 5. Therefore, the total scores ranged from 35 to 175. Using the Thorpe study in 2015 with a criterion point of 92 and rock curve analysis, the RRI cut-off point in this study was 102. Therefore, people who scored higher than 102 have more resources in retirement, and those who scored below have fewer resources. An outstanding AUC, high sensitivity, and high specificity are present at this cut-off point [41]. Youden's J index (0.492) and K-index (0.279), together with the DF index (0.2), indicate the desirability and superiority of this cut-off point (Table 6). Youden's J index is a coefficient that maximizes the sensitivity and specificity of the cut-off point [43]. These indicators are the evaluation criteria of the determined cut-off point. For example, the closer the J index is to 1 and the closer the K and DF indices are to zero, the more appropriate the determined cut-off point is for the statistical population under study [44].

## 4. Discussion

The impact of retirement resources on retirees is crucial, yet it has been overlooked in Iran due to the lack of suitable assessment tools. To fill this gap, we translated and validated the Retirement Resources Inventory (RRI) into Persian. Its psychometric properties and factor structure were evaluated, demonstrating its validity and reliability for use in Iran. This emphasizes the importance of ensuring instrument validity across different cultural contexts.

The Iranian sample's questionnaire comprised 35 items and eight factors: physical, financial, structural social support, functional social support, emotional, cognitive, personal agency, and self-control resources. The structure obtained in our study was similar to the original questionnaire, with the difference that Leung and Earl included physical and financial resources in one factor. However, there are differences in the social and mental subfactors compared to the original study [17]. Our study initially identified two subfactors within mental resources, but secondary factor analysis revealed four subfactors: emotional, cognitive, personal agency, and self-control. Social resources in our study were categorized into two factors: structural and functional.

In contrast, Amorim et al. conducted a Brazilian validation using confirmatory factor analysis and identified a 29-item, five-factor model, including physical health, financial, social, emotional, and cognitive and emotional resources [45]. Similarly, França et al. reported that the Portuguese validation resulted in a well-fitted model comprising 23 items and four factors: physical and financial aspects, social and family resources, received support, and emotional and cognitive resources [46].

These variations in factor structures across different cultural contexts highlight the flexibility of the Retirement Resources Inventory (RRI) in capturing culturally specific dimensions of retirement resources, while also reflecting common underlying constructs. This comprehensive comparison underscores the importance of considering cultural nuances in the validation process and contributes to a deeper understanding of retirement resources globally.

The results indicate that physical and mental resources, as two factors of the identified structure, are extensively researched in retirement studies. Findings underscore the importance of addressing health-related needs in retirement planning [47–49]. Within the mental factor, our study identified four subfactors: emotional, cognitive, personal agency, and self-control.

Emotional resources, such as positive emotions and emotional intelligence, play a critical role in retirement adjustment by enhancing satisfaction and aiding in stress coping among retirees [17]. Empirical evidence suggests that positive emotions improve both physical and mental health, which are crucial for a fulfilling retirement [50]. The broaden-and-build theory by Fredrickson explains that positive emotions expand cognitive and behavioral capacities, leading to greater resilience and well-being [51], which is particularly beneficial during retirement transitions.

Cognitive factors encompass two types: adaptive cognitions (self-esteem, mastery, and optimism) and cognitive functioning (memory, processing speed, learning, and problem-solving ability). Studies have shown that higher self-esteem and a sense of mastery help retirees manage the challenges of retirement, maintaining their mental health and overall life satisfaction [52,53]. Cognitive functioning is vital for daily activities and quality of life, although retirement can have a modest negative effect on cognitive abilities, making the support of these functions through resources and activities essential [54].

Personal agency allows retirees to have control over their retirement decisions, such as the timing and manner of leaving their professional roles. Research indicates that a strong sense of personal agency contributes to smoother role transitions and higher retirement satisfaction [55]. Theoretical frameworks, such as Bandura's self-efficacy theory [56], emphasize that belief in one's ability to exert control over life events enhances motivation and well-being, making personal agency a crucial factor in retirement adjustment.

Self-control resources enable retirees to manage their behaviors and emotions effectively, fostering a sense of control over their retirement journey. A lack of control is a significant indicator of reduced well-being and adjustment problems, highlighting the importance of self-control in navigating retirement challenges [57]. Self-determination theory [58] supports this by suggesting that autonomy and control are fundamental psychological needs that, when met, lead to greater well-being and life satisfaction.

Financial resources, including postretirement income, a sense of income adequacy [17], and planning skills, are necessary for a good retirement course. When facing unforeseen costs after retirement, retirement with limited savings or assets is likely to be difficult [59].

Social resources, known as social support, are categorized into two subdimensions: structural and functional social support resources. Structural support, such as network size and frequency of interaction, and functional support, such as perceived adequacy of relationships, play complementary roles in influencing retirees' well-being and adjustment. A larger, frequently engaged social network (structural support) provides more opportunities for social interaction, reducing loneliness and isolation [60]. These interactions enhance the exchange of material, informational, and emotional support, crucial for coping with retirement challenges [61]. Functional support focuses on the quality of social interactions, reflecting a retiree's satisfaction with their relationships. High-quality functional support significantly enhances mental health, life satisfaction, and overall well-being [62]. Retirees with supportive and satisfying relationships experience lower stress and depression, leading to better retirement adjustment [63]. The interaction between structural and functional support is key to well-being. A large network can provide diverse functional support, but the perceived quality of these relationships determines their effectiveness. For instance, frequent interactions within a large network may still leave a retiree feeling unsupported if the interactions are of poor quality [64]. Conversely, even a small network can be highly beneficial if relationships are strong and supportive [65].

To enhance social support systems for retirees, initiatives should focus on increasing social interactions (structural support) and improving interaction quality (functional support). Community programs facilitating social gatherings, support groups, and volunteer opportunities can expand social networks. Additionally, providing resources on effective communication and relationship-building can help retirees develop deeper, more supportive relationships.

Empirical evidence supports that comprehensive social support systems, addressing both structural and functional aspects, promote retirees' well-being and adjustment [66]. Interventions like peer support programs and community engagement activities improve mental health outcomes and life satisfaction among retirees [67]. By fostering both the quantity and quality of social interactions, these programs help retirees navigate retirement challenges more effectively, leading to better overall well-being.

Uncovering the hidden determinants of human behavior provides a more complete understanding of the causes of performance, especially the use of resources for retirement adjustment. It will also help categorize audiences with common behavioral patterns and identify ways to intervene [68, 69]. Therefore, specific interventions or programs aimed at enhancing retirement resources, such as financial literacy workshops, social support networks, and cognitive-behavioral interventions targeting self-control and personal agency, tailored to the needs of Iranian retirees be suggested.

### 4.1. Strengths and Limitations

This study has several strengths, including the comprehensive validation of the Persian version of the RRI, which provides a robust tool for enhancing retiree health in Persian-speaking communities. The identification of additional subfactors deepens our understanding of retirement resources and their impact on adjustment, facilitating targeted interventions. Moreover, establishing the questionnaire's cut-off point for the first time adds to its practical utility.

However, the study has limitations, including a sample that may not fully represent the diversity of Persian-speaking retirees and the impact of the COVID-19 pandemic on data collection, which affected responses and generalizability. Social restrictions led to validation among retirees with moderate literacy, internet access, and smartphone use, excluding some illiterate retirees. Additionally, the lack of a criterion questionnaire and performing both exploratory and confirmatory factor analysis on the same sample are limitations, suggesting these should be addressed in future studies for comprehensive validation.

Longitudinal studies to examine the stability of retirement resources over time and their impact on long-term retirement outcomes, as well as cross-cultural comparisons to explore cultural variations in retirement experiences and resource utilization, be suggested.

## 5. Conclusion

This study showed that the Persian version of the RRI has favorable psychometric properties. Therefore, the provision of this tool facilitates the development of research and practice in the field of improving the health of retirees at the national level, as well as international comparative research.

## Figures and Tables

**Table 1:** Comparing the mean scores of retirement resources according to sociodemographic variables (*n*=335).

Sociodemographic variables	*N* (%)	Total mean score of retirement resources		
		Mean (SD)	Test of significance	Effect size
*Age (in years)* <60 60–70 >70	76 (22.7) 180 (53.7) 79 (23.6)	112.78 (16.16) 110.05 (14.17) 109.94 (17.13)	*F*=0.94 *P*= .389	0.006

*Gender* Male Female	179 (53.4) 155 (46.3)	109.30 (15.38) 112.24 (15.27)	*t*=−1.74 *P*=0.041^∗^	−0.192

*Marital status* Single Married Other cases	53 (15.8) 270 (80.6) 12 (3.6)	112.60 (16.78) 110.38 (15.24) 107.75 (11.16)	*F*=0.68 *P*=0.507	0.004

*University* SUMS SU MUI UI	102 (30.4) 75 (22.4) 107 (31.9) 51 (15.2)	112.09 (14.47) 109.02 (16.25) 110.02 (14.74) 111.41 (17.11)	*F*=0.67 *P*=0.565	0.006

*Educational level* Primary school Middle school High school Associate degree Bachelor Master degree and higher	13 (3.9) 17 (5.1) 66 (19.7) 69 (20.6) 87 (26) 83 (24.8)	110.84 (17.96) 104.58 (14.50) 106.62 (13.49) 108.13 (16.11) 113.49 (15.81) 114.15 (14.31)	*F*=3.38 *P*=0.005^∗^	0.049

*Job* Academic staff in health Academic staff in nonhealth Administrative Support services Nurse and midwife Public health professional Health care assistant	46 (13.7) 26 (7.8) 112 (33.4) 48 (14.3) 21 (6.3) 36 (10.7) 22 (6.6)	111 (13.48) 118.23 (15.64) 110.25 (16.11) 106.68 (14.66) 114.14 (13.27) 110.72 (14.60) 106.50 (16.48)	*F*=2.10 *P*=0.053	0.040

*Current work* Yes No	68 (20.3) 264 (78.8)	113.55 ± 14.12 109.88 ± 15.67	*t*=−1.75 *P*=0.040^∗^	0.239

*Duration since retirement (years)* <5 5–10 >10	65 (19.4) 98 (29.3) 172 (51.3)	112.63 ± 17.03 112.92 ± 14.97 108.59 ± 14.71	*F*=3.20 *P*=0.042^∗^	0.019

*Type of retirement* Optional Mandatory	122 (36.4) 213 (63.6)	111.87 ± 15.26 109.93 ± 15.42	*t*=1.11 *P*=0.134	0.126

*Number of household members* 1-2 3-4 ≥5	101 (30.1) 195 (58.2) 39 (11.6)	114.01 ± 16.53 109.32 ± 14.15 108.51 ± 17.02	*F*=3.58 *P*=0.029^∗^	0.021

*Note*. ^∗^Significant *P* ≤ 0.05; *F*=one-way ANOVA test; *t* = student *t*-test.

**Table 2:** RRI factor structure consisting of six factors and 35 items (*n* = 335).

Item	F1	F2	F3	F4	F5	F6	M	SD	Test of significance^∗∗^ between two genders
(27). How well are you able to understand and solve problems?	0.75						3.49	0.80	0.009
(28). How well are you able to perform good decision-making?	0.70						3.42	0.83	0.02
^∗^(24). How well are you able to remember the meanings and spellings of different words/concepts?	0.70						3.69	0.87	0.000
(26). In general, how do you assess your speed of processing information?	0.69						3.18	0.85	0.09
(25). How well are you able to acquire new knowledge or skills?	0.68						3.22	0.97	0.30
(21). In general, to what extent do you feel that you have the ability to use emotions to facilitate your thoughts and communication?	0.60						3.25	0.84	0.19
(23). How well are you able to recall events that happened a while ago?	0.58						3.56	0.89	0.38
(20). How much do you have knowledge about how emotions vary or influence behavior?	0.56						3.34	0.89	0.005
^∗^(34). When I cannot progress in something, it is hard for me to find a new approach/way	0.55						3.31	1.11	0.09
(19). How well can you perceive your/others' emotions accurately?	0.50						3.43	0.85	0.27
(18). How much do you experience positive emotions?	0.41						3.16	1.01	0.24
^∗^(35). I create a lot of problems for myself because I set unrealistic goals	0.38						3.74	1.04	0.92
(30). I feel that I am a valuable person, at least on an equal plane with others	0.38						4.02	0.79	0.25
(22). How often do you forget things in the immediate past or where you have placed things?	0.38						3.57	0.91	0.44
^∗^(29). I have little control over the things that happen to me	0.35						3.35	1.11	0.06
(12). To what extent do you consider interaction with your friends supportively?		0.79					2.74	1.01	0.97
(9). How many friends do you have whom you can interact with regularly?		0.68					2.91	0.89	0.77
(11). How many people do you know from various groups in the community?		0.65					2.65	1.03	0.69
(14). To what extent do you consider interactions with acquaintances from various groups in the community supportively?		0.57					2.15	1.08	0.57
(13). To what extent do you consider interaction with your family members supportively?		0.42					3.48	1.04	0.44
(10). How many family members do you have whom you can interact with regularly?		0.39					3.21	0.87	0.61
(6). How much do you have financial support from your personal savings?			0.88				1.95	0.97	0.96
(7). How much do you have financial support from your investments?			0.79				1.74	0.91	0.86
(5). How much is your income to support your/your family's living expenses?			0.59				2.52	0.76	0.03
(8). How much does the superannuation fund support you financially?			0.32				1.78	0.92	0.09
(1). How do you assess your general health status?				0.71			3.36	0.87	0.76
^∗^(2). How much do you suffer from one or more physical illnesses?				0.59			3.57	1.04	0.49
^∗^(3). How much do you suffer from one or more mental disorders?				0.47			4.23	0.92	0.66
(4). How much energy do you have to carry out daily activities or activities that you are interested in?				0.42			3.24	0.90	0.34
(16). To what extent do you receive emotional support from others?					0.71		2.65	0.93	0.56
(17). To what extent do you receive tangible support from others?					0.56		2.17	1.03	0.08
(15). To what extent do you receive informational support from others?					0.52		2.43	0.97	0.84
(32). Even when the situation seems hopeless, I keep fighting to reach my goals						0.68	4.04	0.85	0.40
(31). When faced with problems, I usually increase my efforts						0.58	4.16	0.71	0.82
(33). I can easily adapt to changes in my goals, plans and living conditions						0.55	3.88	0.92	0.76
% eigenvalue: 56.67 % variance: 46.68									

*Note*. ^∗^Items with inverted factorial loads. F1 = mental resources; F2 = structural social support resources; F3 = financial resources; F4 = physical resources; F5 = functional social support resources; F6 = mental resources. Tabachnick and Fidell (2001) recommended a factor loading of 0.32 or higher to be significant [39]. ^∗∗^Independent *t*-test.

**Table 3:** Mental factor analysis.

Item	F1 (Emotional resources)	F2 (Cognitive resources)	F3 (Personal agency resources)	F4 (Self-control resources)
Q26	0.63			
Q24	0.62			
Q27	0.60			
Q25	0.57			
Q28	0.55			
Q23	0.49			
Q22	0.32			
Q19		0.68		
Q20		0.62		
Q21		0.62		
Q18		0.56		
Q32			0.83	
Q33			0.64	
Q31			0.61	
Q30			0.30	
Q35				0.63
Q34				0.59
Q29				0.34

*Note*. Extraction method: unweighted least squares. Rotation method: equamax with Kaiser normalization^a^. ^a^Rotation converged in 9 iterations.

**Table 4:** The final structure of RRI including its subfactors and related items.

Factor	Subfactor	Range of score	Items
Physical	—	4–20	Q1, Q2, Q3, Q4

Financial	—	4–20	Q5, Q6, Q7, Q8

Social	Structural social support	6–30	Q9, Q10, Q11, Q12, Q13, Q14
	Functional social support	3–15	Q15, Q16, Q17

Mental	Emotional	4–20	Q18, Q19, Q20, Q21
	Cognitive	7–35	Q22, Q23, Q24, Q25, Q26, Q27, Q28
	Personal agency	4–20	Q30, Q31, Q32, Q33
	Self-control	3–15	Q29, Q34, Q35

**Table 5:** The goodness of fit indices for RRI's eight-factor model.

Model	*χ* ^2^/df	TLI	CFI	GFI	IFI	RMR	RMSE
Model (8 factor)	1.95	0.87	0.88	0.85	0.88	0.05	0.05

*Note*. *χ*^2^(df): chi-square statistics (degree of freedom), TLI: Tucker–Lewis index, CFI: comparative fit index, GFI: goodness of fit index, RMR: root mean square residual, RMSEA: root mean square error of approximation.

**Table 6:** AUC value, sensitivity, and specificity of ROC curve for RRI.

Scale	AUC	95% CI		*p* value	Cut-off point	Sensitivity	1-Specificity	Yuden's J	K-index	DIFF
		Lower bound	Upper bound							
RRI	0.664	0.541	0.786	0.04	102	1	0.683	0. 492	0.279	0.2

*Not*e. *p* ≤ 0.05; AUC = area under curve; CI = confidence interval; *D* value or K-index = Sqrt [(1 − sensitivity)^2^ + (1 − specificity)^2^; DIFF = abs (sensitivity − specificity) [42].

## Data Availability

The data that support the findings of this study are available from the corresponding author upon reasonable request.
